# Setting up an Intra‐Arterial Chemotherapy Center in a Low‐Resource Country in Sub‐Saharan Africa

**DOI:** 10.1002/puh2.70434

**Published:** 2026-11-06

**Authors:** Vera Adobea Essuman, Benjamin Dabo Sarkodie, Dziffa‐Bella Imelda‐Odille Ofori‐Adjei, Vera Mawusime Beyuo, Andrea Nimako, Abigail Nyarko Bosompem, Lauretta Pinamang Owusu‐Asare, Yaw Akye Essuman, Benjamin Abaidoo, Fred Kwabena Osei, Dong Jatoe, Lorna Awo Renner, Naa Naamuah Tagoe, Kwesi Nyan Amissah‐Arthur

**Affiliations:** ^1^ Lions International Eye Center Korle Bu Teaching Hospital Accra Ghana; ^2^ Ophthalmology Unit, Department of Surgery University of Ghana Medical School Accra Ghana; ^3^ Department of Radiology University of Ghana Medical School Accra Ghana; ^4^ Department of Anaesthesia Korle Bu Teaching Hospital Accra Ghana; ^5^ Department of Child Health Korle Bu Teaching Hospital Accra Ghana; ^6^ St. Thomas Eye Hospital Accra Ghana

**Keywords:** Ghana, globe salvage, intra‐arterial chemotherapy, low‐resource setting, retinoblastoma

## Abstract

**Background:**

Intra‐arterial chemotherapy (IAC) has shown promise in improving outcomes for patients with Retinoblastoma (RB) globally. Creating a safe, effective, and sustainable IAC center in Ghana, a country with a high RB burden, will provide access to much‐needed specialized treatment for patients with RB.

**Aim:**

This study aims to outline key considerations, obstacles, and opportunities for setting up an IAC center in a low‐resource country.

**Methods:**

This narrative implementation report describes the establishment of an IAC service through a KBTH–Euracare partnership. Service requirements, barriers, and costs were identified from routine planning meetings, site reviews, stakeholder consultations, supplier information, and early implementation experience, informed by international centers and adapted locally. The model combines Euracare's infrastructure with trained KBTH personnel, mentorship, and knowledge transfer. Lessons reflect a descriptive review of implementation decisions and challenges.

**Findings:**

Cost analysis revealed that the most viable strategy would be a phased implementation, beginning with a public–private partnership anchored by a major tertiary hospital to ensure scalability and resource optimization. Potential enablers include training and skills transfer, existing radiology units, and collaboration from international partners to support capacity‐building initiatives. Key barriers to establishing an IAC center in Ghana include lack of trained specialists, high equipment costs, and financial constraints.

**Conclusion:**

Establishing an IAC center in a low‐resource country requires careful planning, coordination, and resource allocation. Despite the challenges, there are opportunities for partnerships, technology transfer, task‐shifting, research, and innovation.

## Graphical abstract

Ghana established West Africa's first intra‐arterial chemotherapy service for retinoblastoma through a public–private partnership between Korle Bu Teaching Hospital and Euracare, delivering 32 treatment sessions for 10 children. Strategic partnerships, training, and phased implementation can make this vision‐ and life‐saving therapy achievable in low‐resource settings.

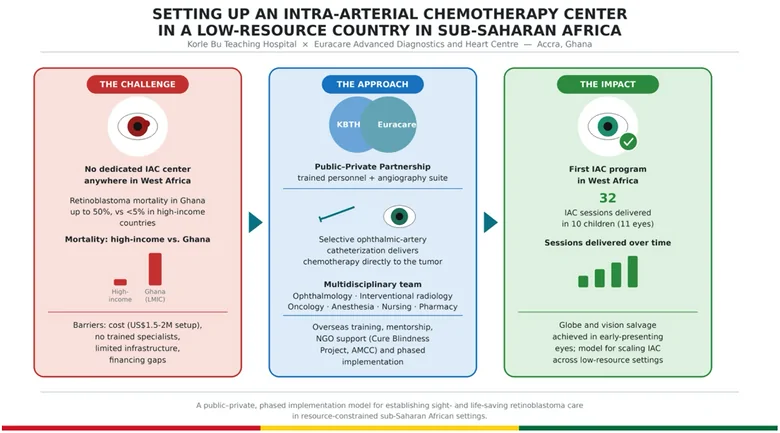

## 1 Introduction

Retinoblastoma (RB) is the most common intraocular cancer in childhood [1]. Although survival is high (>95%) in developed countries, factors such as late presentation due to lack of awareness of the seriousness of the disease, financial constraints, and traditional beliefs reduce the chance of survival in low‐ and middle‐income countries (LMICs) such as Ghana [2, 3]. Mortality from RB ranges between 40% and 70% in most developing/low‐income countries [4], with a 50% rate observed in Ghana [3]. The disparity in morbidity and mortality is also partly due to differences in treatment options available to patients, especially those with bilateral disease [5]. In high‐income countries, genetic studies are incorporated into the routine workup for RB [6]. This helps to determine prognoses concerning the risk of recurrence and secondary cancers [4]. Intra‐arterial chemotherapy (IAC), intravitreal chemotherapy, laser, and other focal therapies allow useful vision to be salvaged in eyes with RB that present early, especially in bilateral cases where one eye may have been enucleated [7]. IAC, in particular, may be useful for groups B‐D disease stages, especially when the tumor size is greater than 3‐disc diameters (DD) [8].

The cost of setting up such facilities is prohibitive, and in many LMICs, it is considered a low priority, as many pressing issues compete for the same restricted resources. Before 2017, Ghana's premier health facility, the Korle Bu Teaching Hospital (KBTH), which manages most RB cases in and outside the country and has trained personnel for IAC, lacked the facility, justifying its establishment to enhance local, specialized pediatric oncology care. Since Ghana does not yet have a dedicated IAC center, one must be established. Significant obstacles prevent children with RB from receiving treatment overseas, such as difficult visa procedures, exorbitant expenses, and a lack of financial assistance. By removing these obstacles, cutting down on treatment delays, and ensuring fair access to life‐saving care, a local center would ease the financial and psychological strain on families while bolstering the capacity of pediatric oncology nationwide. Training of health personnel to provide these services also poses a challenge, as equipment and facilities are not available. Thus, setting up a modern and comprehensive RB treatment center in LMICs usually requires public–private partnerships.

Ghana is the first country in West Africa to set up an IAC center and the second after South Africa in Sub‐Saharan Africa (SSA). The purpose of this narrative implementation report is to chronicle the journey to setting up an IAC facility in Ghana and to share the lessons learnt throughout the process.

## 2 Methods

### 2.1 Local Setting

Ghana has a population of 30.8 million, comprising 35.3% children aged less than 15 years [9]. There are approximately 90 ophthalmologists in active service [9]. Even though the ophthalmologist‐to‐population ratio is approximately 1:333,000, that is, 80% of the WHO target (1:250,000), only four pediatric ophthalmologists are serving the whole pediatric population. The majority of ophthalmologists are concentrated in the major cities, and many rural areas are greatly underserved [9]. Until IAC was introduced, treatment modalities available in Ghana for the treatment of RB were: systemic chemotherapy, intravitreal chemotherapy, transpupillary thermotherapy (TPTT), focal laser photocoagulation, cryotherapy, surgery (enucleation), and external beam radiation therapy (EBRT). RB, along with three other childhood cancers (acute lymphoblastic leukemia, Burkitt lymphoma, and Wilms tumor), was only recently covered by the National Health Insurance Scheme (NHIS), with the cost of some treatment modalities being absorbed [10].

### 2.2 The Study Design

This was a narrative implementation report describing the planning and early establishment of an IAC service in Ghana, a resource‐constrained setting. It draws on routine activities by clinical, administrative, infrastructure, financing, and service‐delivery teams at KBTH and the Euracare Advanced Diagnostics and Heart Centre (Euracare).

Capacity and service requirements were documented through planning meetings, site reviews, and consultations with personnel involved in RB care, interventional radiology, and program delivery. Reviews covered infrastructure, equipment, consumables, workforce, training, financing, and care pathways, informed by established international IAC centers and adapted to local resources.

Barriers were identified from stakeholder discussions, institutional and site reviews, and challenges encountered during planning and early implementation. Costs were estimated from infrastructure, equipment, consumables, training, and operational requirements, supplemented by supplier and institutional information. Funding options were reviewed, and a KBTH–Euracare public–private partnership was adopted.

Under this model, trained KBTH personnel provide specialized IAC services at Euracare, using the latter's interventional radiology infrastructure. Training, mentorship, and knowledge transfer support local capacity and sustainability. Lessons were derived from the implementation team's review of planning decisions, adaptations, barriers, costs, and early operational experience and are presented descriptively, not as findings from formal qualitative or quantitative analysis.

### 2.3 The Implementation Pathway for Setting up IAC in Ghana

The development of a comprehensive IAC program for RB in Ghana commenced in 2017 under the leadership of the Lead Pediatric Ophthalmologist (V.A.E) at KBTH. To provide a clear and reproducible account of this process, Table 1 presents the principal implementation milestones, spanning from the initial needs assessment in 2017 through the treatment of the first patient in April 2020 and the continuation of services to date. Each stage is delineated by the specific actions undertaken, the personnel and partner institutions engaged, the infrastructure and consumables mobilized, the funding and cost arrangements secured, and the key operational challenges encountered.

**TABLE 1:** Sequential implementation pathway for intra‐arterial chemotherapy (IAC) for retinoblastoma in Ghana.

Sequence/period	Implementation stage	Key action	Staff/partners	Infrastructure, equipment, or supplies	Funding/cost arrangement	Main difficulty or practical consideration
1. 2017	Initial needs assessment and program initiation	Leadership at Korle Bu Teaching Hospital identified the need for a comprehensive local IAC service for retinoblastoma and assessed gaps in specialist skills, infrastructure, and treatment access	Lead pediatric ophthalmologist and institutional stakeholders	Assessment of neurointerventional capability, angiography access, medicines, consumables, and patient‐care pathways	No dedicated start‐up funding was available	Government facilities lacked the equipment required for IAC
2. 2017	Identification of the interventional radiologist	An interventional radiologist with foundational neurointerventional expertise was identified and engaged by the lead pediatric ophthalmologist to perform IAC and subsequently train others. The need for the IAC and his role in the retinoblastoma treatment team was discussed	Interventional radiologist at Korle Bu Teaching Hospital; lead pediatric ophthalmologist	Existing neurointerventional knowledge and access to future specialist training	Training support to be mobilized	Local experience in selective ophthalmic‐artery catheterization was limited
3. 2017	Selection of the catheterization laboratory. Figures 1 and 2	Euracare Advanced Diagnostics and Heart Centre in Accra was selected because it had the infrastructure required for arterial catheterization. The interventional radiologist recommended this center, led the pediatric ophthalmic team to engage the management of Euracare. A case was strongly made for Euracare to support the initiative as part of its Corporate Social Responsibility	KBTH team and Euracare staff	Private angiography catheterization laboratory and associated procedural equipment	Private‐facility partnership used to avoid the immediate cost of building a new laboratory	No government institution had suitable catheterization equipment
4. 2018	Overseas training	The selected interventional radiologist undertook refresher training at Apollo Hospitals in Hyderabad, India, gaining advanced skills in ophthalmic‐artery catheterization	Interventional radiologist; Apollo Hospitals trainers; Himalayan Cataract Project, now Cure Blindness Project	Specialist training environment, angiography facilities, and catheterization materials	Training of the interventional radiologist was funded by the Cure Blindness Project	Specialized training was unavailable locally and required overseas placement
5. 2019	Creation of the multidisciplinary team	A retinoblastoma team was constituted to coordinate patient selection, anesthesia, treatment, drug handling, imaging, nursing care, follow‐up, and program evaluation	Pediatric ophthalmologists, vitreoretinal surgeons, pediatric oncologists, anesthesiologists, ophthalmic nurses, pharmacists, imaging technicians, and researchers	Existing staff and departmental resources in both KBTH and Euracare	Phased use of existing personnel minimized recruitment costs	Coordinating several disciplines without overburdening staff or interrupting other services
6. 2019 onward	Funding arrangements and service partnership	A non‐profit partnership with Euracare was formalized to provide infrastructure and consumables. NGOs, corporate entities, and philanthropic organizations were engaged for training and equipment, consumables and medicines	Korle Bu, Euracare, Cure Blindness Project, Alliance Mondiale Contre Le Cancer, corporate, and philanthropic partners	Catheterization laboratory, donated equipment, procedural consumables, and chemotherapeutic agents	IAC was initially paid for by patients; longer term incorporation into the National Health Insurance Scheme was anticipated	Dependence on external support and out‐of‐pocket payment threatened affordability and sustainability
7. Pre‐treatment phase	Pharmacy and supply‐chain preparation	Pharmacists and private pharmaceutical partners organized access to chemotherapeutic agents, including melphalan, and prepared for safe, timely treatment‐day supply	Pharmacists, pediatric oncology team, and private pharmaceutical companies	Chemotherapeutic agents, preparation facilities, handling procedures, and reliable supply arrangements	Corporate or philanthropic support was sought for medicines; patients initially bore treatment costs	Maintaining an affordable and consistent supply of specialized drugs
8. Pre‐treatment phase	Referral pathways and patient selection	Clear pathways were established for timely specialist referral, multidisciplinary assessment and selection of patients for IAC when focal therapies or prior systemic treatment were inadequate	Referring clinicians, pediatric ophthalmologists, pediatric oncologists, interventional radiologist, and the wider multidisciplinary team, usually done MDT weekly meetings	Clinical assessment, ocular imaging, treatment‐planning, and communication systems	Delivered through existing services and the developing program	Ensuring timely referral, coordinated decision‐making and continuity of care
9. April 2020	First treated patient	A 21‐month‐old child with bilateral retinoblastoma received the first IAC session in Ghana using melphalan, topotecan, and carboplatin. The procedure was successful and uneventful; three sessions were delivered at four‐week intervals	Interventional radiologist and the multidisciplinary retinoblastoma team	Catheterization laboratory, angiography and catheterization equipment, chemo drugs, anesthesia, and peri‐procedural support	Implemented under the established partnership and initial patient‐payment model	Delivering a new procedure safely while coordinating specialized personnel, medicines, consumables, and follow‐up

### 2.4 Practical Timeline for Establishing the IAC Program

**FIGURE 1:**
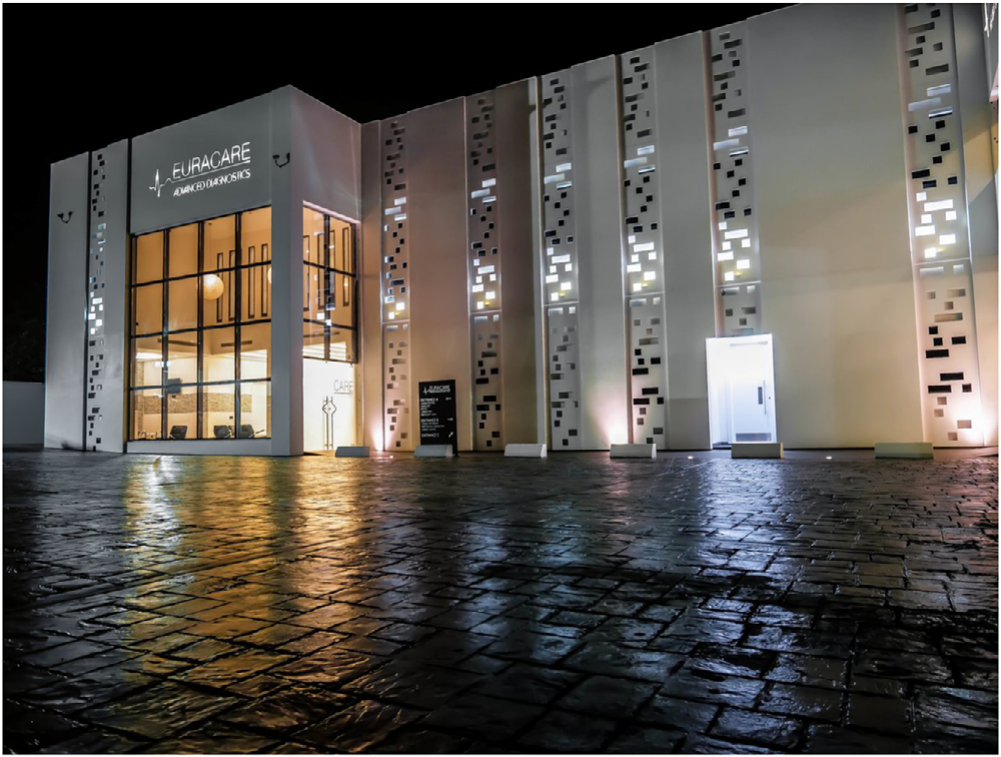
Euracare Advanced Diagnostics and Heart Centre, a private cardiac facility, with the necessary infrastructure, identified to provide intra‐arterial chemotherapy services in Ghana.

**FIGURE 2:**
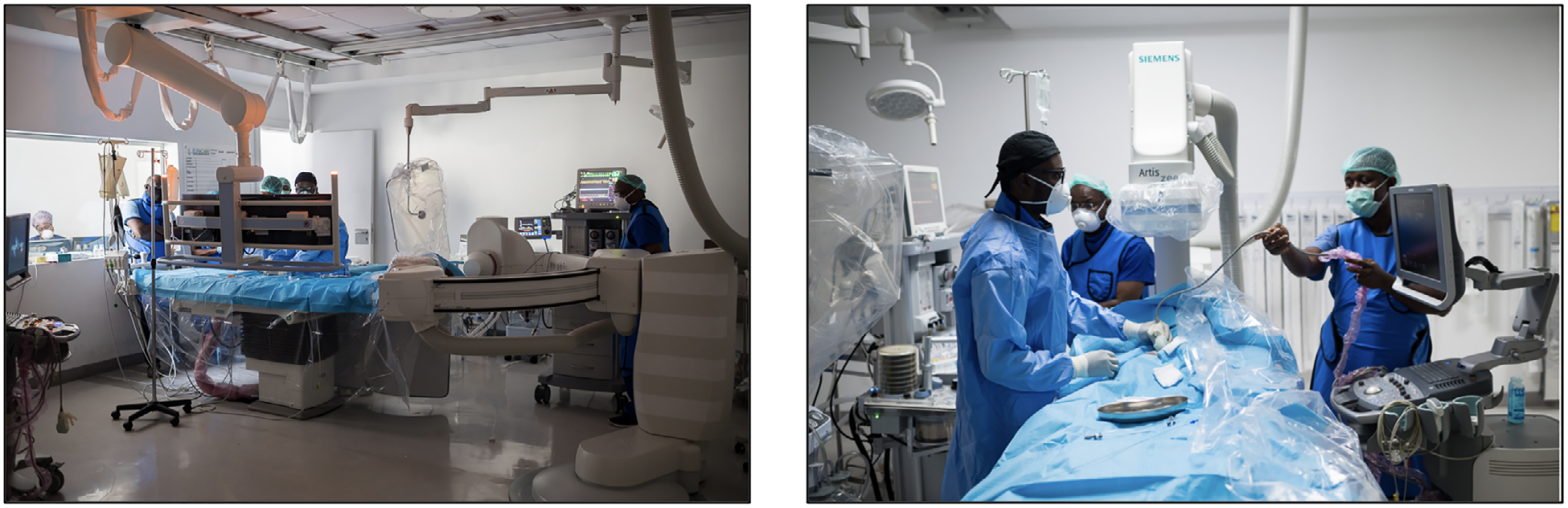
The Cardiac catheterization laboratory setup for intra‐arterial chemotherapy procedures.

### 2.5 How the Multidisciplinary Team Works During IAC Sessions

Before IAC, each child undergoes an examination under anesthesia at KBTH, including fundus imaging, tumor mapping, and documentation, with focal laser photocoagulation or cryotherapy performed when indicated. Intravitreal chemotherapy may also be administered as required. On the scheduled IAC day at Euracare, the pediatric ophthalmologist conducts a repeat examination under anesthesia using an indirect ophthalmoscope with a 20D lens and scleral indentation to reassess tumor status and confirm the treatment plan. The pharmacist prepares the chemotherapeutic agents, after which the interventional radiologist performs selective ophthalmic artery catheterization and administers the drugs. Throughout the procedure, the anesthesiologist maintains anesthesia and monitors the patient, while the pediatric oncology team and technicians coordinate patient preparation, intra‑operative support, and recovery. Post‑procedure, the pediatric oncology and anesthesia teams monitor for early complications and assess readiness for discharge.

Three weeks after IAC, that is, 1 week before the next scheduled session, the pediatric ophthalmology team re‑examines the child under anesthesia at KBTH to evaluate tumor response and provide focal therapy if feasible. At present, Euracare integrates the pre‑treatment examination under anesthesia and IAC into a single session, whereas diagnostic assessment, imaging, tumor documentation, systemic therapy, focal laser photocoagulation, cryotherapy, and intravitreal chemotherapy are generally performed separately at KBTH. Greater integration of clinically compatible procedures has the potential to reduce repeated anesthesia exposure, minimize travel demands, lower treatment‑related costs, and lessen disruption to families.

### 2.6 The Initiation of IAC in Ghana

The first beneficiary of IAC in Ghana was a 21‐month‐old female diagnosed with bilateral RB at the age of 11 months. She had enucleation of the right eye following six cycles of systemic chemotherapy with Vincristine, Etoposide, and Cyclophosphamide. Her left eye had multiple tumor foci, with the largest being 10 DD in size. TPTT and cryotherapy were, thus, not suitable focal therapies for such a large peripheral tumor. She was thus recommended for IAC. Her first session was done in April 2020, using Melphalan. The procedure was successful and uneventful. She had three sessions, each spaced 4 weeks apart. (Table 2).

**TABLE 2:** IAC sessions and chemotherapeutic drugs used for the index patient in Ghana.

Date	Session number	Drug(s) used
April 2020	1	Melphalan
May 2020	2	Melphalan + Topotecan
June 2020	3	Melphalan + Topotecan + Carboplatin

The tumor subsequently shrank, allowing TPTT to be performed after the third session of IAC (Figure 3). Her last post‐treatment best corrected visual acuity was 6/9, using the Cardiff Acuity Tests. To date, a total of 32 sessions of IAC have been done in Ghana.

**FIGURE 3:**
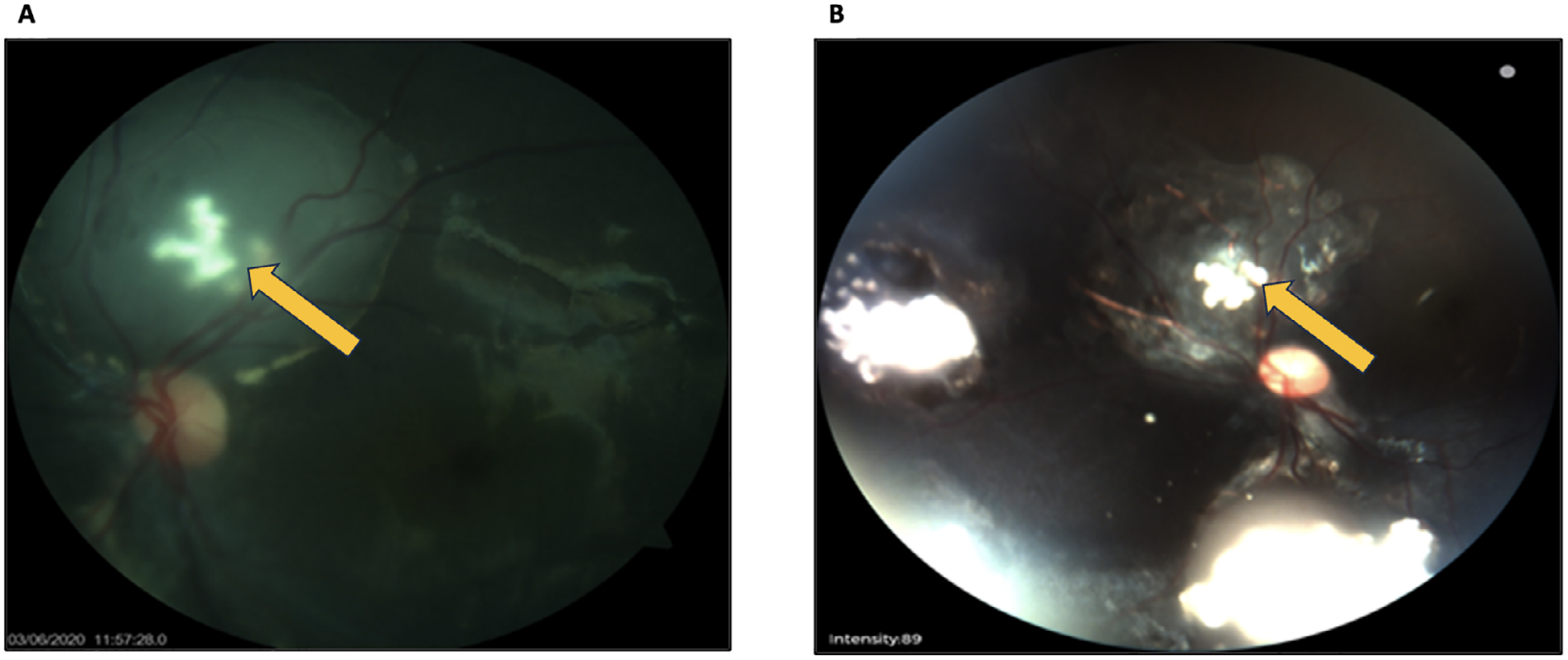
Management of retinoblastoma in the index case with intra‐arterial chemotherapy. (A) Pre‐treatment fundus image with a yellow arrow indicating an active lesion with massive subretinal fluid. (B) Post‐treatment fundus image showing predominantly type 4 regression of the active lesion (flat scar) and resolved sub‐retinal fluid.

### 2.7 The Continuation of the IAC Service to Date

Since the introduction of IAC in Ghana in April 2020, 32 treatment sessions have been conducted in 10 children, involving 11 eyes. In all cases, IAC was administered as secondary therapy. Two children had unilateral disease; the remaining eight had bilateral RB. Out of these eight children with bilateral disease, one had both eyes treated, while the other seven underwent IAC in the second eye following enucleation of the fellow eye. The current protocol consists of a triple‐drug regimen of melphalan, carboplatin, and topotecan administered during each IAC session.

### 2.8 Challenges Encountered

1. Funding: Funding was the main challenge encountered throughout all stages of the process. Funding was required for the procurement of initial software and other essential equipment, training of the interventional radiologist, and the purchase of consumables and the cost of the process. The initial cost of setting up such facilities for IAC, including a Cath lab, in LMICs is prohibitive (1.5‐2 million USD) [11], as well as recurring expenses (catheters, drugs). Limited health insurance coverage exacerbates patient affordability issues. Intra‑arterial chemotherapy is not currently covered under Ghana's NHIS, requiring families to finance treatment privately. Many rely on charitable donations or individual contributions, often mobilized through media appeals supported by formal letters from pediatric ophthalmologists at KBTH. Children who meet the clinical criteria for IAC but are unable to secure funding undergo enucleation of the affected eye. As a result, IAC is generally restricted to patients with bilateral disease in whom one eye has already been enucleated or deemed unsalvageable, and to selected cases of early‑stage unilateral disease where families can afford or successfully raise the necessary funds.
2. Drug preparation and preservation: Initial challenges with drug preparation and preservation were encountered, as proper storage and preservation of these drugs require strict temperature and time controls. Melphalan has a very short half‐life and needs to be prepared promptly for the procedure to prevent wastage [12]. In the early stages, determining the exact time the drug would be required was difficult, resulting in significant wastage. However, over time and with practice, this hurdle has been overcome.
3. Distance between the two centers: The necessity of coordinating treatment between the two separate hospitals presented logistical challenges. Transporting patients, drugs, and specialized personnel sometimes resulted in delays and increased costs. Currently, patients scheduled for IAC are admitted to Euracare on the day of surgery and discharged home following the procedure. Those traveling from outside Accra, or without family support in the city, are admitted to KBTH for pre‑ and post‑operative care surrounding the intervention.
4. Infrastructure: IAC requires specialized equipment, including angiography suites, chemotherapy pumps, and trained personnel. Low‐resource countries often lack the necessary infrastructure, including modern hospitals, equipment, and trained staff.
5. Training and expertise: IAC requires a highly skilled, multidisciplinary team with specialized training and expertise in interventional radiology, pediatric oncology, anesthesiology, and nursing. Low‐resource countries often face shortages of skilled healthcare professionals. The lack of trained specialists in the country necessitated international collaborations and training, which are costly and time‐consuming.
6. Resource constraints: This is a resource‐intensive treatment that requires significant investment in equipment, personnel, and consumables. Governments of low‐resource countries often struggle to allocate resources for specialized cancer treatments.
7. Regulatory frameworks: Absence of clear national guidelines for IAC may complicate implementation and quality control. Low‐resource countries may lack regulatory frameworks and standards for IAC, making it challenging to establish and maintain a safe and effective treatment program.
8. Infrastructure deficits: At project inception, public health facilities across the country were markedly deficient in the infrastructure required for IAC, lacking angiography suites, chemotherapy preparation units, and a stable electricity supply, thus necessitating public–private partnerships. The challenge was further exacerbated by difficulties in maintaining sensitive equipment in environments prone to dust and humidity.
9. Workforce shortages: A scarcity of interventional radiologists, oncologists, and specialized nursing staff hampers service delivery. The existing healthcare workforce experiences heavy workloads, resulting in burnout and reduced efficiency. Ongoing brain drain and insufficient training programs further perpetuate these gaps.
10. Patient access and awareness: Low awareness of RB among the general public and even healthcare providers contributes to delays in presentation and diagnosis [13]. Many parents do not recognize early signs of the disease, resulting in presentation at advanced stages [14]. Geographic and financial barriers further prevent families from seeking timely specialist care [15]. Cultural beliefs and stigma around cancer and poor health literacy also hinder early presentation and contribute to treatment hesitancy [16].

### 2.9 Opportunities

Despite the challenges, several opportunities have emerged in the process of establishing an IAC unit for the treatment of RB in Ghana, a resource‐constrained sub‐Saharan African country. These opportunities provide a foundation for sustainable growth and improved patient outcomes.

1. Partnerships and collaborations: Establishing strategic partnerships with international oncology centers and non‐governmental organizations (NGOs) can facilitate knowledge transfer, training, and technical support. Regional collaborations between neighboring low‐resource countries allow for shared expertise, pooling of resources, and cross‐border referrals for specialized care.
2. Technology transfer: Introducing new technologies, such as portable angiography suites, can improve access to IAC in low‐resource settings. The adoption of digital imaging and telemedicine platforms has enabled remote consultation and diagnosis with international experts, reducing delays in treatments. Eye equipment donations such as retinal cameras and binocular indirect ophthalmoscopes for diagnosis and monitoring of treatment outcomes, as well as capacity‐building initiatives, have introduced cutting‐edge technologies and skills to local healthcare institutions.
3. Task‐shifting and training: Task‐shifting and training programs can help build local capacity and expertise in IAC, reducing reliance on external experts. Training of ophthalmologists, pediatric oncologists, and radiologists in IAC techniques can expand the pool of healthcare professionals capable of delivering specialized care. The development of structured training programs for healthcare providers can enhance supportive care and post‐treatment monitoring. Collaboration with medical institutions for residency and fellowship programs in pediatric ophthalmology, pediatric oncology, and interventional radiology is fostering long‐term capacity building. Task‐shifting to trained mid‐level healthcare workers has allowed for early screening and referral, improving early‐stage detection and patient outcomes.
4. Research and innovation: Establishing a national RB registry has enabled better data collection, epidemiological studies, and policy planning for pediatric ophthalmology and pediatric oncology. Low‐resource countries can participate in research studies and innovation initiatives to develop new IAC protocols, technologies, and strategies tailored to their specific needs. Innovation in low‐cost drug preparation and preservation methods has enhanced the affordability and sustainability of IAC treatment.

Table 3 presents a comparison of IAC setups in selected developing countries (upper‐ and low‐ middle‐income countries) around the world, including Colombia [17], India [18], Pakistan [19], and Argentina [20, 21].

**TABLE 3:** Comparison of IAC setups in selected developing countries.

Country	Institution name	Type of institution	Funding model	Number of patients	Training pathway	Implementation challenges
Ghana	Korle Bu Teaching Hospital partnered with Euracare Advanced Diagnostics and Heart Centre	Tertiary ophthalmology referral center and a private cardiac facility	Implementation relied on public–private partnership, NGO support (Cure Blindness Project, Alliance Mondiale Contre Le Cancer), and patient out‑of‑pocket payments	11 eyes (10 patients)	Ophthalmologists specialized in pediatric ophthalmology and retina collaborated with interventional neuroradiologists. Training is through institutional fellowship programs and international mentorship, adapting techniques to local infrastructure	Funding and infrastructure deficits
Colombia	Hospital San Vicente Fundación/Universidad de Antioquia, Medellín	Public teaching hospital and ophthalmology referral center	Mixed: primarily private nonprofit foundation with patient care funded through a combination of direct payment, insurance coverage, and institutional support	85 patients (100 eyes)	Ophthalmologists specialized in pediatric retina and ocular oncology collaborated with interventional neuroradiologists. Training was through institutional fellowship programs and international mentorship, adapting techniques to local infrastructure	Late disease presentation, insurance‑related delays, concentration of expertise in a single center, treatment complexity, and technical risks
India	Sankara Nethralaya/Medical Research Foundation, Chennai	Tertiary oncology and ophthalmology referral center	Predominantly private nonprofit/charitable hospital model, with patient care funded through a mix of direct payment, insurance, institutional and NGO subsidies	15 (15 eyes)	Ophthalmologists trained in ocular oncology and pediatric retina collaborated with interventional neuroradiologists. Training was through institutional fellowship programs and hands‑on angiography suite experience	High cost and technical demands of IAC, requiring specialized infrastructure and expertise. The study also highlights the absence of pharmacies for drug compounding
Pakistan	Lahore General Hospital, Lahore	Public teaching hospital	Charitable nonprofit cross‐subsidy model	3 eyes	Ophthalmologists collaborated with interventional radiologists; training was through institutional oncology fellowship programs and angiography suite practice, with adaptation of international protocols to local resources	High cost and limited infrastructure for IAC. The study highlights financial barriers, scarcity of specialized equipment, and concentration of expertise in a single center as major obstacles to wider adoption
Argentina	Hospital Juan P. Garrahan (HPG) and Clinica y Maternidad Suizo Argentina, Buenos Aires	Public national tertiary pediatric center	Costs are entirely borne by the Argentine governmental agencies and health insurance companies	97 eyes (81 patients)	Pediatric ophthalmologists collaborated with pediatric oncologists and a neurosurgeon; with training sponsored by the Fund for Ophthalmic Knowledge set up by a twinning program between HPG and Memorial Sloan Kettering Cancer Center, New York. This program was established in 1995 and has resulted in the significant increase in clinical and research capacity at HPG [21].	Lack of consistent access to some chemotherapeutic agents including Melphalan, and a lack of available equipment at the primary site, HPG

## 3 Discussions and Lessons Learned

The establishment of an IAC unit for the treatment of RB in a resource‐constrained Sub‐Saharan African country has highlighted key challenges and opportunities that can inform future initiatives.

Ghana, being the first West African country to set up an IAC center, has demonstrated that it is possible for resource‐constrained countries, such as ours, to provide this highly specialized and much‐needed service to help in the fight against RB and save the lives and vision of affected children. The current population of West Africa at the time of this study was about 417,871,618 [22]. Ghana's single IAC center was the only one providing IAC services in all of West Africa at the time. Currently, the KBTH has acquired a new cardiac catheterization laboratory (Cath lab), which can provide IAC. Hopefully, this new setup in a public institution will reduce transfer time for patients between the two centers: KBTH and Euracare Diagnostic Centre (about 9 km apart). It will also strengthen resident training in the public hospital.

The disproportionately high burden of RB in developing countries [23], such as Ghana, makes it essential for all treatment modalities, such as IAC, to be made available to improve outcomes. In addition, as developing countries make attempts to change the narrative of late presentation via awareness creation campaigns and advocacy, treatment modalities for early disease, including IAC, should be available and accessible to patients, especially since, in these cases, globe salvage and, in some instances, vision preservation are achieved.

Regarding referral, travel and continuity of follow‐up for children requiring IAC, children referred from Ghana and neighboring West African countries are assessed at the Eye Clinic of KBTH. To streamline referrals and minimize delays, referring ophthalmologists are encouraged to contact the center through dedicated telephone lines before the child's arrival. Caregivers are counselled on the proposed treatment, expected duration and frequency of procedures, and the importance of adhering to scheduled follow‐up. During the intensive treatment phase, families travelling from distant locations may stay at the KBTH Mothers’ Hostel, near the Department of Child Health, which provides heavily subsidized accommodation; families able to manage repeated travel may return home between sessions and attend according to a predetermined schedule. Nevertheless, transport and accommodation expenses, loss of caregiver income, missed work, and prolonged separation from other family members remain substantial barriers that may lead to delayed treatment, missed appointments, or treatment abandonment. Follow‐up appointments are scheduled and communicated by the treating team. When an appointment is missed, the team and pediatric oncology social workers attempt to contact the caregiver, identify barriers, reschedule the visit, and, where necessary, conduct a home visit. For children residing outside Ghana, the team collaborates with referring ophthalmologists to support adjunctive treatment and long‐term follow‐up locally. Although this cross‐border follow‐up model is not yet standardized for local children, the forthcoming National Retinoblastoma Strategy and Treatment Guideline is expected to address existing gaps in referral and continuity of care.

A prominent challenge encountered was funding, which affected infrastructure development, equipment procurement, and the affordability of treatments for patients. A strategic partnership with a private hospital, Euracare, ensured that we were able to use their infrastructure and setup to deliver the service. Played a crucial role in overcoming financial and technical barriers. Despite this support from external partners, the high cost of the procedure for patients remains a barrier for many families. The current cost of one session of IAC per eye under this public–private partnership is USD 5000. A total of at least three cycles is required for treatment, bringing the total cost to between USD 15,000 and USD 30,000 per eye or in bilateral cases, respectively [24]. This is beyond the financial capability of most Ghanaian families, where the minimum wage is between $1 and $2 per day [25]. This financial barrier requires further remedy through advocacy for governmental support for the setting up of infrastructure in public tertiary facilities, which will likely reduce costs for patients. The cost of IAC services varies across countries worldwide, with lower costs reported in India (USD 1000–2000), while more advanced countries such as China (USD 9422 ± 3709 per patient) and the United States (USD 40,000) have higher per‐session costs [26]. IAC is not currently covered by Ghana's NHIS, and the limited state subsidy for RB treatment generally does not meet the full cost of treatment. Although the NHIS supports clinical assessment, coordination, personnel, and available institutional services, families must finance the actual cost of IAC. Those unable to pay may seek philanthropic support through medical appeal letters issued by health facilities, which outline the child's diagnosis, clinical need, and estimated treatment cost. These appeals, often supported by pediatric ophthalmologists at KBTH and publicized through the media, solicit contributions from individuals, charitable organizations, corporate bodies, diaspora networks, and international service organizations. While such support has enabled some children to receive treatment, it remains unpredictable and may delay or prevent access. Consequently, clinically eligible children who cannot secure funding may undergo enucleation. Access to IAC in our setting is often limited to children with bilateral disease when one eye has already been enucleated or is considered unsalvageable, and to selected early‐stage unilateral cases whose families can afford or successfully raise the required funds. In the short term, a blended financing model combining a centrally managed philanthropic fund or foundation, corporate and charitable partnerships, and appropriately structured interest‐free instalment or microfinance arrangements may improve access. Ultimately, expanding the NHIS to cover IAC and comprehensive RB care would provide the most sustainable and equitable financing mechanism, reduce dependence on ad hoc fundraising, and ensure that treatment decisions are based on clinical need rather than ability to pay.

RB1 genetic testing is currently unavailable in Ghana. Consequently, children presenting with bilateral disease are presumed to have heritable RB, prompting routine sibling screening. Pediatric ophthalmologists and oncologists provide counseling on the elevated risk of second malignancies, while children with cancer, particularly those admitted to KBTH, receive psychosocial support from a multidisciplinary team comprising a child psychologist, social workers, and a Child Life Specialist. The Ghana Parents’ Association for Childhood Cancer (GHAPACC) further supplements this care by offering family support. Collectively, these stakeholders foster continuity of care and help families navigate concerns related to treatment, enucleation, repeated procedures, financial hardship, and uncertainty surrounding survival and vision outcomes.

The experiences of Ghana, India, Colombia, Argentina, and Pakistan in establishing IAC centers for RB reveal both shared struggles and context‑specific adaptations. In all five countries, the cost of infrastructure and consumables emerged as the most formidable barrier, with Ghana and Pakistan particularly constrained by prohibitive per‑session expenses relative to average household income [19]. India's Sankara Nethralaya leveraged charitable and cross‑subsidy models [18]. At the same time, Colombia's Hospital San Vicente Fundación relied on a private nonprofit foundation with insurance support, highlighting how institutional funding structures shaped patient access [17]. Argentina's Hospital Juan P. Garrahan (HPG) covered all related treatment costs through governmental support and health insurance coverage [20, 21]. Training pathways also differed: Ghana depended on international fellowships and tele‑mentoring, India emphasized institutional subspecialty fellowships, Colombia combined local fellowship training with international mentorship, and Pakistan adapted oncology fellowship structures within a cancer hospital [17, 18, 19]. The HPG in Argentina has a twinning program with the Memorial Sloan Kettering Cancer Center in New York, which has ensured international training experiences in clinical and research work [21]. HPG now serves as an international referral and training center for many South American nations [21]. Despite these differences, all programs required close collaboration between ophthalmologists and interventional radiologists, underscoring the multidisciplinary nature of IAC. Implementation challenges centered on advanced disease presentation, concentrated expertise in single centers, and limited public health insurance coverage, which restricted scalability. Yet, each country demonstrated that with strategic partnerships, NGO support, and phased implementation, IAC could be introduced in resource‑limited settings, offering hope for globe salvage and vision preservation in children otherwise facing enucleation.

Significant lessons learned were the importance of collaboration, training, and technology transfer. Partnerships with international oncology centers facilitated specialized training, while telemedicine and digital imaging enhanced early diagnosis and remote consultations. Task‐shifting also emerged as an effective approach, enabling trained mid‐level healthcare providers to support early detection and patient management.

Additionally, research and innovation proved essential for adapting IAC protocols to local conditions. The creation of an RB registry has provided valuable epidemiological data, guiding policy and treatment strategies. The development of cost‐effective drug preparation and preservation methods has improved the feasibility of treatment in a low‐resource setting.

Finally, while challenges such as infrastructure limitations, resource constraints, and access remain, this project has demonstrated that with strategic partnerships, capacity building, and innovative approaches, advanced cancer treatments such as IAC can be successfully introduced and sustained in resource‐limited settings. These lessons will lay the foundation for scaling services and improving pediatric oncology care in the region.

## 4 Conclusion

Establishing an IAC center in a low‐resource country requires careful planning, coordination, and resource allocation. Despite the challenges, leveraging strategic interventions, including partnerships, technology transfer, task shifting, and research innovation, significantly enhanced the establishment and sustainability of an IAC Unit in Ghana. By addressing these barriers and following the recommendations outlined in this article, other low‐resource countries can develop effective IAC programs to enhance access to life‐saving treatment and improve outcomes for children with RB in SSA.

The following recommendations outline key strategies for establishing and sustaining intra‐arterial chemotherapy (IAC) services, with a focus on infrastructure development, workforce capacity building, strategic partnerships, quality assurance, and equitable access to treatment:

1. Conduct a needs assessment: Conduct a thorough needs assessment to identify the demand for IAC, available resources, and existing infrastructure.
2. Develop a business plan: Develop a comprehensive business plan, including budget, staffing, and equipment requirements.
3. Establish partnerships: Establish partnerships with international organizations, specialized cancer centers, and industry partners to access expertise, technology, and resources.
4. Provide training and capacity‐building: Provide training and capacity‐building programs for local healthcare professionals, including interventional radiologists, oncologists, and nurses.
5. Develop a quality assurance program: Develop a quality assurance program to ensure the safety and efficacy of IAC treatments.
6. Financing and equitable access: Microfinance options and government subsidies should be pursued to improve affordability. Comprehensive NHIS coverage for RB treatment, supplemented by a dedicated childhood‐blindness fund supported by private, corporate, and philanthropic contributions, would provide a more equitable and sustainable financing model.
7. Build human resource capacity: To strengthen capacity through the training of neurosurgeons and radiologists in IAC techniques, delivered via tele‐mentoring and fellowship programs. To take advantage of RB‐focused initiatives offered by international institutions, such as the International Agency for the Prevention of Blindness (IAPB) and the Eye Cancer Foundation (ECF).
8. To pool regional resources for bulk purchasing of catheters and drugs.
9. To encourage local manufacturing and production of essential supplies (e.g., heparin) to enhance accessibility and cut costs.

## Author Contributions

**Vera Adobea Essuman:** conceptualization, project administration, supervision, writing – original draft, writing – review and editing. **Benjamin Dabo Sarkodie:** conceptualization, data curation, supervision, writing – original draft, writing – review and editing. **Dziffa‐Bella Imelda‐Odille Ofori‐Adjei:** data curation, writing – review and editing. **Vera Mawusime Beyuo:** data curation, writing – original draft, writing – review and editing. **Andrea Nimako:** data curation, writing – review and editing. **Abigail Nyarko Bosompem:** data curation, writing – review and editing. **Lauretta Pinamang Owusu‐Asare:** data curation, writing – review and editing. **Yaw Akye Essuman:** data curation, project administration, writing – original draft, writing – review and editing. **Benjamin Abaidoo:** data curation, formal analysis, writing – original draft, writing – review and editing. **Fred Osei:** data curation, writing – review and editing. **Dong Jatoe:** data curation, writing – review and editing. **Lorna Awo Renner:** conceptualization, writing – review and editing. **Naa Naamuah Tagoe:** data curation, writing – review and editing. **Kwesi Nyan Amissah‐Arthur:** conceptualization, supervision, writing – review and editing.

## Funding

The Himalayan Cataract Project (now Cure Blindness Project) provided funding for overseas training of the interventional radiologist; however, they did not have any role in the design of the study, collection, analysis and interpretation of data, and writing of the report. Neither did they influence the decision to publish this article.

## Ethics Statement

The study was carried out in accordance with Article 2.5 of the Tri‐Council Policy Statement for Ethical Conduct of Research.

## Conflicts of Interest

The authors declare no conflicts of interest.

## Data Availability

Data sharing is not applicable to this article, as no datasets were generated or analyzed during the current study.
